# Identification of the Solid Stem Suppressor Gene *Su-TdDof* in Synthetic Hexaploid Wheat Syn-SAU-117

**DOI:** 10.3390/ijms241612845

**Published:** 2023-08-16

**Authors:** Hui Li, Xin Liu, Junqing Zhang, Longyu Chen, Minghu Zhang, Yongping Miao, Pan Ma, Ming Hao, Bo Jiang, Shunzong Ning, Lin Huang, Zhongwei Yuan, Xuejiao Chen, Xue Chen, Dengcai Liu, Hongshen Wan, Lianquan Zhang

**Affiliations:** 1 State Key Laboratory of Crop Gene Exploration and Utilization in Southwest China, Sichuan Agricultural University, Chengdu 611130, China; 13197298301@163.com (H.L.);; 2 Triticeae Research Institute, Sichuan Agricultural University, Chengdu 611130, China; 3 Key Laboratory of Wheat Biology and Genetic Improvement on Southwestern China (Ministry of Agriculture and Rural Affairs), Crop Research Institute, Sichuan Academy of Agricultural Sciences, Chengdu 610066, China; 4 Environment-Friendly Crop Germplasm Innovation and Genetic Improvement Key Laboratory of Sichuan Province, Chengdu 610066, China

**Keywords:** semisolid, gene mapping, *Aegilops tauschii*, synthetic hexaploid wheat, lodging resistance

## Abstract

Lodging is one of the most important factors affecting the high and stable yield of wheat worldwide. Solid-stemmed wheat has higher stem strength and lodging resistance than hollow-stemmed wheat does. There are many solid-stemmed varieties, landraces, and old varieties of durum wheat. However, the transfer of solid stem genes from durum wheat is suppressed by a suppressor gene located on chromosome 3D in common wheat, and only hollow-stemmed lines have been created. However, synthetic hexaploid wheat can serve as a bridge for transferring solid stem genes from tetraploid wheat to common wheat. In this study, the F_1_, F_2_, and F_2:3_ generations of a cross between solid-stemmed Syn-SAU-119 and semisolid-stemmed Syn-SAU-117 were developed. A single dominant gene, which was tentatively designated *Su-TdDof* and suppresses stem solidity, was identified in synthetic hexaploid wheat Syn-SAU-117 by using genetic analysis. By using bulked segregant RNA-seq (BSR-seq) analysis, *Su-TdDof* was mapped to chromosome 7DS and flanked by markers *KASP-669* and *KASP-1055* within a 4.53 cM genetic interval corresponding to 3.86 Mb and 2.29 Mb physical regions in the Chinese Spring (IWGSC RefSeq v1.1) and *Ae. tauschii* (AL8/78 v4.0) genomes, respectively, in which three genes related to solid stem development were annotated. *Su-TdDof* differed from a previously reported solid stem suppressor gene based on its origin and position. *Su-TdDof* would provide a valuable example for research on the suppression phenomenon. The flanking markers developed in this study might be useful for screening *Ae. tauschii* accessions with no suppressor gene (*Su-TdDof*) to develop more synthetic hexaploid wheat lines for the breeding of lodging resistance in wheat and further cloning the suppressor gene *Su-TdDof*.

## 1. Introduction

Wheat is the largest cereal crop in the world, accounting for 220 million hectares with annual global production of 772 million tons [1]. The world’s population is expected to increase by nearly 2 billion over the next 30 years [2], and there will be greater demand for global wheat production. Therefore, we must strive to increase wheat yield. However, with the continuous improvement of wheat yield and the increase in fertilizer application, lodging has become an important factor affecting the high and stable yield of wheat [3,4,5,6,7]. Plant dwarfing can effectively alleviate the lodging damage of wheat [8]; however, severe dwarfism leads to inadequate biomass accumulation and lower yield potential [9]. Increasing the mechanical strength of the stem is another breeding strategy [3]. The ratio of stem wall thickness to stem diameter and the content of mechanical tissue in solid-stemmed wheat were found to be significantly higher than those in common wheat [10,11,12]. Therefore, compared with common wheat, solid-stemmed wheat has higher stem strength and lodging resistance, which play an important role in wheat lodging resistance breeding [11].

To date, two candidate genes, *TraesCS3B01G608800* and *TRITD3BV1G280530*, have been reported for stem solidity in hexaploid wheat and durum wheat, respectively. The differentially expressed gene *TraesCS3B01G608800* (KAF7034036.1) showed copy number variations associated with stem solidity in different hexaploid wheat cultivars [13]. However, *TRITD3BV1G280530* was confirmed as a candidate gene in *SSt1* in durum wheat, and the copy numbers of *TRITD3BV1G280530* in solid-stemmed and hollow-stemmed durum wheat were different. The protein encoded by *TRITD3BV1G280530* is a zinc finger protein, and its physical location is 829.1 Mb on chromosome 3BL of the durum wheat genome v1.0 [14]. Liu et al. [15] found that a QTL for pith thickness in wheat was previously discovered on 3BL in a double haploid population of ‘Westonia’ × ‘Kauz’. A putative vacuolar processing enzyme gene *TaVPE3cB* was screened out as a potential pith-thickness candidate gene in Australian ‘Westonia’ wheat [15].

There are many solid-stemmed varieties, landraces, and old varieties in durum wheat (*Triticum turgidum* L. ssp. *durum*, AABB, 2n = 4x = 28) [16]. Durum wheat has greater stem firmness and more stable genetic characteristics than those of common wheat varieties [17,18,19]. Beginning in the 1940s, breeders tried to transfer the solid stem gene from Golden Ball to hexaploid wheat by crossing, but the solid stem trait was suppressed, and only hollow-stemmed lines were created [17,20,21]. Then, it was found that the expression of the solid stem gene was suppressed, and the suppressor gene was presumed to be located on chromosome 3D in common wheat [17]. Suppression is a common phenomenon in nature. For example, suppression of disease resistance is especially frequent in the expression genes for resistance to fungal plant pathogens causing stem rust, leaf rust, and powdery mildew [22,23,24,25,26,27,28]. Inhibition often occurs, especially during the transfer of foreign genes from diploid and tetraploid ancestors to hexaploid wheat [22,23,24,27,29,30].

Synthetic hexaploid wheat was an effective bridge for transferring the solid stem gene from durum wheat to hexaploid wheat. A synthetic hexaploid wheat (P89-77-1 F_4_) derived from crosses between solid-stemmed Golden Ball durum wheat and *Ae. squarrosa* L. expressed pith in the culm lumen [31]. Then, two solid-stemmed hexaploid spring wheat lines (PI 633,737 and PI 633,738) were developed and released by backcrossing P89-77-1 F_4_ with the hollow-stemmed hexaploid wheat cultivar AC Elsa [31,32]. In our previous study, the expression of a solid second internode at the base of the stem was stable for two synthetic hexaploid wheat lines (Syn-SAU-117 and Syn-SAU-119), which were developed from a cross of the semidwarf solid-stemmed durum wheat line Ma as the female parent and two different *Ae. tauschii* accessions (AS92 and AS96) as the male parent [33]. The second internode at the base of the stem of Syn-SAU-117 (pedigree: Ma × AS92) was semisolid, while that of Syn-SAU-119 (pedigree: Ma × AS96) was solid in both the greenhouse and field. It was indicated that the D genome of *Ae. tauschii* AS92 suppressed the expression of the solid stem gene from the 3B chromosome in Syn-SAU-117 [33]. The objective of this study was to identify and map the solid stem suppressor gene in Syn-SAU-117 by using bulked segregant RNA-seq (BSR-seq) analysis.

## 2. Results

### 2.1. Differential Expression of the TdDof Gene in Different Materials

**Figure 1:**
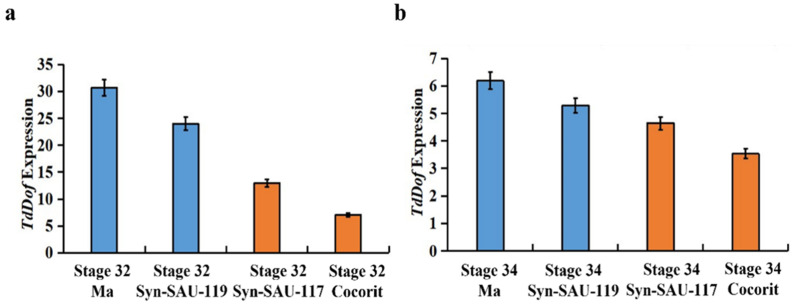
The expression differences of the *TdDof* gene in Ma, Syn-SAU-119, Syn-SAU-117, and Cocorit at Zadoks Stage 32 and Zadoks Stage 34: (**a**) expression differences in the *TdDof* gene at the Zadoks Stage 32; (**b**) expression differences in the *TdDof* gene at the Zadoks Stage 34.

The gene expression levels of *TdDof* were determined in two synthetic wheat cultivars, Syn-SAU-117 and Syn-SAU-119, and two durum wheat cultivars, Ma (solid) and Cocorit (hollow), as solid- and hollow-stemmed contrasts during early internode elongation (Zadoks stage 32 and Zadoks stage 34). The expression level of *TdDof* was higher in Syn-SAU-119 than in Syn-SAU-117 in both periods (Figure 1a,b).

### 2.2. Genetic Analysis of the Solid Stem Suppressor Gene Su-TdDof

**Figure 2:**
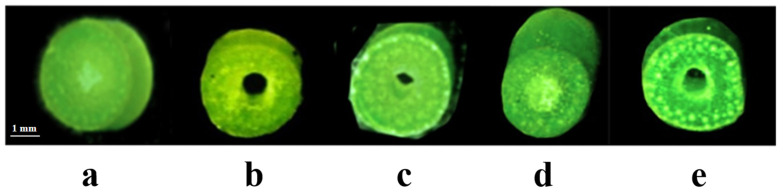
The stem solidity of Syn-SAU-119 and Syn-SAU-117 and the individual plants of F_1_ and F_2_ in the greenhouse: (**a**) Syn-SAU-119; (**b**) Syn-SAU-117 × Syn-SAU-119 F_1_; (**c**) Syn-SAU-117; (**d**) solid F_2_ plant; (**e**) semisolid F_2_ plant.

**Figure 3:**
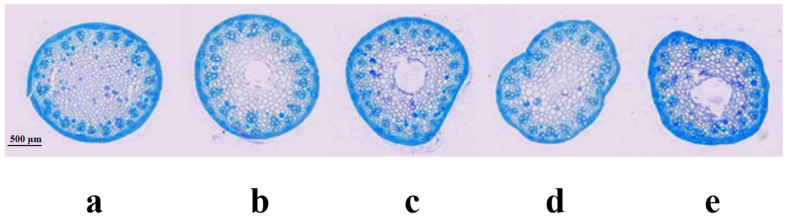
Anatomical structure of the stems of Syn-SAU-119 and Syn-SAU-117 and individual plants of F_1_ and F_2_ in the greenhouse: (**a**) Syn-SAU-119; (**b**) Syn-SAU-117 × Syn-SAU-119 F_1_; (**c**) Syn-SAU-117; (**d**) solid F_2_ plant; (**e**) semisolid F_2_ plant.

According to Pauw et al. [34], synthetic hexaploid wheat Syn-SAU-119 was solid-stemmed (solidity = 5.0; on 1–5 scale) (Figure 2a), and Syn-SAU-117 was semisolid-stemmed (solidity = 4.2; on 1–5 scale) (Figure 2c). Syn-SAU-117 and Syn-SAU-119 were hybridized to obtain F_1_, F_2_, and F_2:3_ populations for the genetic analysis of solid stem inhibition in Syn-SAU-117. The solidity of stems of all F_1_ plants was similar to that of Syn-SAU-117 plants (solidity = 4.0) (Figure 2b). The F_2_ population was segregated into 36 solid-stemmed (solidity = 5) and 120 semisolid-stemmed (1 < solidity < 5) plants, fitting a 1S: 3S_S_ ratio (*χ*^2^ = 0.308, *p* = 0.579) (Figure 2e) (Table 1), indicating that the inhibition of solid stems was conferred by a single dominant gene that was tentatively designated as *Su-TdDof*. At Zadoks stage 34, the anatomical structure of the stalks of the synthetic hexaploid wheat showed that the Syn-SAU-119 parenchyma was complete (Figure 3a), and the parenchyma of Syn-SAU-117 and Syn-SAU-117 × Syn-SAU-119 F_1_ showed a similar degree of apoptosis (Figure 3b,c). In the Syn-SAU-117 × Syn-SAU-119 F_2_ population, the parenchyma of some plants was complete (Figure 3d), while that of other plants showed apoptosis (Figure 3e). The segregation rate of the F_2:3_ population composed of 134 families was 35 (homozygous solid): 67 (heterozygous): 32 (homozygous semisolid) (*χ*_1:2:1_^2^ = 0.134, *p* = 0.935), which was consistent with the segregation results for the F_2_ population (Table 1).

### 2.3. BSR-Seq Analysis of the RNA of Bulks with Contrasting Stem Solidity

**Figure 4:**
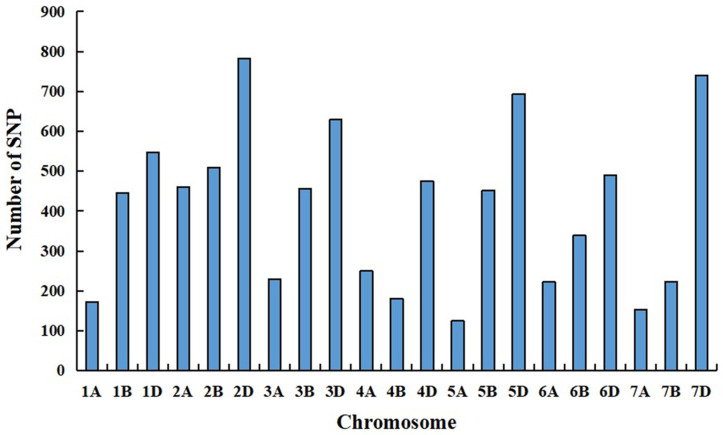
Distribution of SNPs (AFD > 0.6, *p-value* < 1 × 10^−8^) on 21 chromosomes.

**Figure 5:**
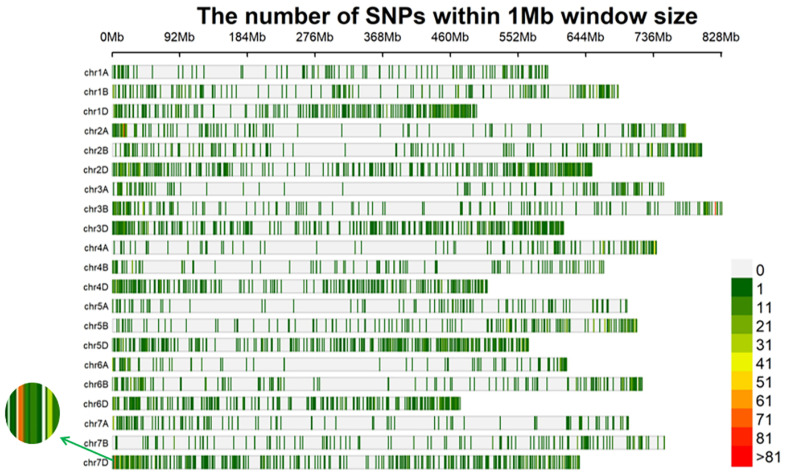
The enrichment of SNPs within a window size of 1 Mb on wheat chromosomes. The green arrow indicates the high-density SNP enrichment that occurred on chromosome 7DS.

The RNA samples of the solid bulk and the semisolid bulk were subjected to RNA-seq analysis, which generated 56,746,340 and 82,341,750 raw reads, respectively. After quality control, 56,740,782 and 82,334,750 high-quality reads from the solid bulk and semisolid bulk, respectively, were uniquely mapped to the Chinese Spring genome (IWGSC RefSeq v1.1). A total of 8581 SNPs (*p* <  1 × 10^−8^ and AFD > 0.6) were identified from these reads by using the GATK v4.0 software (Figure 4). One hundred and twenty-three SNPs were located in an 8 Mb genomic interval (4–11 Mb) on the short arm of chromosome 7D in the Chinese Spring genome (IWGSC RefSeq v1.1), and these were regarded as candidate SNPs linked to *Su-TdDof* (Figure 5).

### 2.4. Molecular Mapping of Su-TdDof

**Figure 6:**
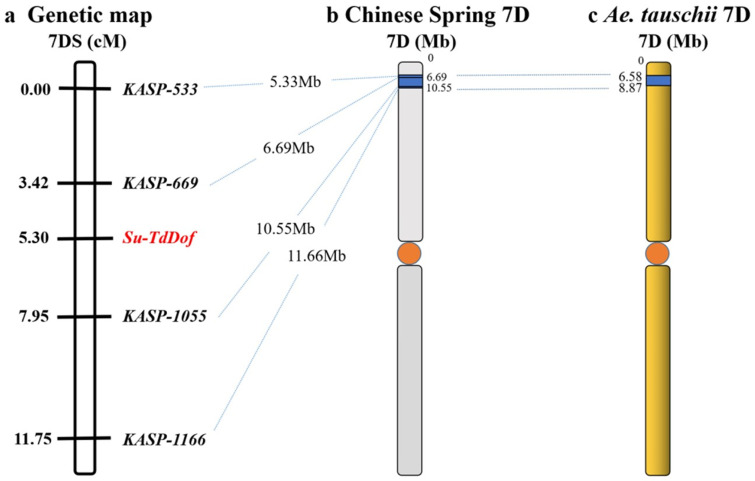
Genetic linkage map of the *Su-TdDof* gene on chromosome 7DS showing the physical location of *Su-TdDof*: (**a**) linkage map of *Su-TdDof*; (**b**) the physical interval (blue part) where the four KASP markers linked to *Su-TdDof* were anchored in Chinese Spring, with orange dots represent centromeres, dotted lines indicating the physical positions of each marker; (**c**) physical intervals anchored by markers linked to *Su-TdDof* in *Aegilops tauschii*.

Fifty-one out of the 123 clustered SNPs on 7DS were chosen to develop KASP markers. Four of them were successfully converted into KASP markers (*KASP-533*, *KASP-669*, *KASP-1055*, *KASP-1166*) (Table 2) and scored reliably on the parents, as well as the solid and semisolid bulks. All tested markers exhibited identical haplotypes between Syn-SAU-117 and its male parent AS92 but were distinct from those of Syn-SAU-119 and its male parent AS96 (Table 3). Subsequently, these KASP markers were used to genotype 134 F_2_ plants derived from the cross between solid-stemmed Syn-SAU-119 and semisolid-stemmed Syn-SAU-117 plants. Linkage analysis indicated that *KASP-669* was potentially mapped 1.88 cM distally, and *KASP-1055* was placed 2.65 cM proximally to *Su-TdDof* (Figure 6).

### 2.5. Gene Analysis of the Genomic Region of Su-TdDof

**Figure 7:**
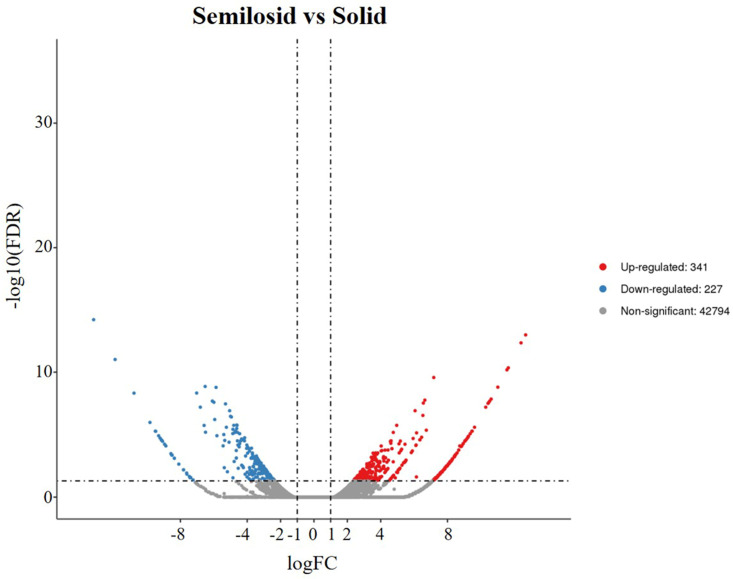
Volcano map of differentially expressed genes.

The sequences of the closely linked markers *KASP-669* and *KASP-1055* were blasted against the Chinese Spring genome and the *Ae. tauschii* genome to obtain their physical positions. *Su-TdDof* was physically mapped to a 3.86 Mb region between the 6.69 Mb and 10.55 Mb regions of the Chinese Spring 7DS chromosome (IWGSC RefSeq v1.1) and between the 6.58 Mb to 8.87 Mb regions (2.29 Mb) in the *Ae. tauschii* AL8/78 7DS chromosome (*Ae. tauschii* AL8/78 v4.0) (Figure 6). There were 180 and 125 predicted genes in the target physical regions in Chinese Spring and *Ae. tauschii* AL8/78, respectively (IWGSC RefSeq v1.1; *Ae. tauschii* AL8/78 v4.0; Supplementary Tables S1 and S2). In the Chinese Spring genome, ten genes may be associated with the growth and development of plant stems, including six zinc-finger-protein-related genes (*TraesCS7D02G015800*, *TraesCS7D02G019600LC*, *TraesCS7D02G022000LC*, *TraesCS7D02G023300LC*, *TraesCS7D02G023400LC*, *TraesCS7D02G024900LC*), two biofunction inhibitor genes (*TraesCS7D02G020000*, *TraesCS7D02G020100*), one pectin lyase-like superfamily protein gene (*TraesCS7D02G016300*), and one homeobox-like protein BEL1 gene (*TraesCS7D02G019800*). Five genes, including two zinc-finger-protein-related genes (*AET7Gv20034800*, *AET7Gv20042400*), one 36.4 kDa proline-rich protein gene (*AET7Gv20040700*), one transcription factor gene (*AET7Gv20036400*), and one homeobox-like protein BEL1 gene, were found in the *Ae. tauschii* genome, which had a good collinear relationship with those of Chinese Spring (Figure S1). Transcriptome analysis revealed a total of 43,362 differentially expressed genes, including 341 upregulated genes, 227 downregulated genes, and 42,794 nondifferentially expressed genes (Figure 7). There were 12 and 7 significantly differentially expressed genes in the target physical regions in the Chinese Spring genome and *Ae. tauschii* genome (Supplementary Tables S3 and S4, Figure S1). Among them, the annotations of *TraesCS7D02G016300*, *AET7Gv20040000,* and *AET7Gv20040700* were probably associated with the growth and development of plant pith [14,35,36,37,38,39,40,41]. *TraesCS7D02G016300* was a gene encoding pectin lyase superfamily proteins (PG) that acted on pectin and lignin in the cell wall and promoted cell wall degradation and shedding, thus promoting cell apoptosis (Supplementary Table S3). The homologous genes of *TraesCS7D02G016300* were two polygalacturonase genes (*AET7Gv20034200* and *AET7Gv20034900*) in the *Ae. tauschii* genome (*Ae. tauschii* AL8/78 v4.0), and their gene annotations were not correlated with the inhibition of solid stems (Supplementary Tables S3 and S4). *AET7Gv20040700* and *AET7Gv20040000* in the *Ae. tauschii* genome (*Ae. tauschii* AL8/78 v4.0) had a good collinear relationship with those of Chinese Spring (Figure S1). *AET7Gv20040000* was annotated as a homeobox-like protein BEL1 gene (Supplementary Table S4). The homologous gene of *AET7Gv20040000* in Chinese Spring was *TraesCS7D02G019800*, and its functional annotation was a homeobox-like protein BEL1 gene (Supplementary Table S1), which was the same as *AET7Gv20040000* in the *Ae. tauschii* AL8/78 genome. *AET7Gv20040700* was annotated as a 36.4 kDa proline-rich protein gene (Supplementary Table S4). The homologous gene of *AET7Gv20040700* in Chinese Spring was *TraesCS7D02G020100*, and its functional annotation was that of a bifunctional (protease/α-amylase) inhibitor/plant lipid transfer protein/seed storage helical domain (Supplementary Table S1).

## 3. Discussion

To date, lodging is still a problem in wheat-growing regions worldwide, despite scientists having made great efforts to solve it for many years. The selection of excellent germplasms with alternative semidwarf genes or good stem mechanical strength may be an effective way to solve this problem [42]. Solid-stemmed wheat has strong lodging resistance due to its higher stalk strength [11,43]. The pith is composed of undifferentiated parenchymatous cells in the solid stems, which accounted for 11% of the total stem dry weight 10–14 days after anthesis and contributed 13% of ethanol-soluble carbohydrates in the entire stem [44]. Water-soluble carbohydrates (WSCs), including sugars, such as fructans, sucrose, glucose, and fructose, are stored in the stems [45,46], and they could be remobilized and transported to the developing grains [47], thus playing an important role in buffering grain yield [48,49]. Stem solidness was reported to be negatively correlated with yield in a few older reports [50,51]. However, later studies showed no such association of the variation of solid stem traits with a decrease in wheat yield [52]. Moreover, the development of high-yielding solid-stemmed cultivars is not related to the degree of stem solidness [53,54,55,56,57].

Durum wheat has many solid-stemmed varieties, landraces, and old varieties [16]. However, attempts to transfer solid stem genes to hexaploid wheat via direct crossing have been unsuccessful because the expression of solid stem genes is suppressed by the suppressor gene on chromosome 3D in common wheat [10,20,21]. In this study, a new solid stem suppressor gene, *Su-TdDof*, was identified in the synthetic hexaploid wheat Syn-SAU-117 and mapped on chromosome arm 7DS; it was flanked by the markers *KASP-669* and *KASP-1055* within a 4.53 cM genetic interval corresponding to the 3.86 Mb physical region in the Chinese Spring genome (IWGSC RefSeq v1.1). In addition, the expression of the solid stem gene *TdDof* in Syn-SAU-117 was lower than that in Syn-SAU-119, thus confirming the existence of the solid stem suppressor gene *Su-TdDof*.

In common wheat, the existence of many suppressor genes affects the normal expression of some important genes and the utilization of excellent foreign genes [28,43,58,59,60,61,62]. To date, many disease resistance genes and corresponding suppressor genes have been found in the D genome of common wheat and the D genome donor *Ae. tauschii* [43,61,63,64], such as the leaf rust suppressor gene *Su-Lr23* on chromosome 2DS [62] and the stem rust suppressor gene *SuSr-D1* on chromosome 7DL of the hexaploid wheat cultivar ‘Canthatch’ (CTH) [65,66,67,68]. A recent study showed that the gene *SuSr-D1* encoded Med15, a subunit of the Mediator complex that suppressed the expression of stem rust resistance [28]. In the present study, *Su-TdDof* was from *Ae. tauschii*, which was different from the suppressor gene presumed to be located on chromosome 3D in common wheat found by Larson et al. [10].

*Su-TdDof* was physically mapped to the region between 6.58 Mb and 8.87 Mb (2.29 Mb) on the *Ae. tauschii* AL8/78 7DS chromosome (*Ae. tauschii* AL8/78 v4.0) (Figure 6). Based on the gene functional annotation and screening of differentially expressed genes in the transcriptome, there were two protein-coding genes, *AET7G20040000* and *AET7Gv20040700*, in the target physical regions in the *Ae. tauschii* genome (Supplementary Tables S3 and S4, Figure S1). *AET7Gv20040000* was annotated as a homeobox-like protein BEL1 gene, and the homologous gene of *AET7Gv20040000* in Chinese Spring was *TraesCS7D02G019800*. Its functional annotation was the same as that of *AET7Gv20040000*. The genes of the BEL1 protein family play an important role in the growth and development of plant stems, leaves, flowers, and other organs [36,38]. For example, in *Arabidopsis thaliana*, the specific interaction between the BEL1 protein-like family genes BLH6 and KNAT7 inhibits the transcription factor REVOLUTA (REV), affecting growth and development in the stem of Arabidopsis inflorescences and, thereby, regulating secondary cell wall development [37]. *AET7Gv20040700* was annotated as a 36.4 kDa proline-rich protein gene, and the homologous gene of *AET7Gv20040700* in Chinese Spring was *TraesCS7D02G020100*. Its functional annotation was that of a bifunctional (protease/α-amylase) inhibitor/plant lipid transfer protein/seed storage helical domain. Studies have shown that the formation of pith in the stem is related to starch [14,35,39]. *AET7Gv20040700* may inhibit the hydrolysis of starch and affect the formation of pith. These two genes, *AET7Gv20040000* and *AET7Gv20040700*, will be cloned and sequenced in future studies to further develop markers for verification.

During the introduction of foreign genes into common wheat, with the increase in ploidy, the expression of superior genes decreased or was completely inhibited because of the existence of suppressor genes [43,58]. Therefore, exploring new suppressor genes, screening accessions without suppressor genes, or carrying out artificial mutation of suppressor genes can enable breeders to break through this restriction and provide beneficial help for the introduction of foreign genes into common wheat [69]. The flanking markers *KASP-669* and *KASP-1055* developed in this study could be used as molecular markers to screen recombinant heterozygous plants, construct secondary F_2_ populations and develop markers, and further narrow the location interval to finely map and clone *Su-TdDof*. The flanking markers *KASP-669* and *KASP-1055* were also used to screen *Ae. tauschii* accessions with no suppressor gene (*Su-TdDof)* to develop more synthetic hexaploid wheat lines with solid stems for the breeding of lodging resistance. Solid-stemmed synthetic hexaploid wheat can be used as a bridge to cross with elite wheat cultivars [70]. Combined with molecular-marker-assisted selection, the transfer of solid stem genes from tetraploid wheat into common wheat cultivars and the breeding of new wheat cultivars with solid stems will provide new materials for the breeding of lodging resistance in wheat.

## 4. Materials and Methods

### 4.1. Plant Materials

Two synthetic hexaploid wheat lines (Syn-SAU-117 and Syn-SAU-119), two different durum wheats (Ma and Cocorit), and two different *Ae. tauschii* (2n = 2x = 14, DD) accessions (AS92 and AS96) were used in this study. Syn-SAU-117 and Syn-SAU-119 were generated via natural chromosome doubling of Ma × AS92 F_1_ and Ma × AS96 F_1_, respectively. Syn-SAU-117 and Syn-SAU-119 were identified by using FISH with the oligonucleotide probes Oligo-pSc119.2-1 and Oligo-pTa535-1 [33]. Plants with 42 chromosomes were used in this study. The durum wheats Ma (solid-stem) and Cocorit (hollow-stem) were supplied by George Fedak at the Ottawa Research and Development Center for Agriculture and Agri-Food (Ottawa, ON, Canada). The lines with the AS code were stored in our institute. All materials used in this study were kept at the Triticeae Research Institute of Sichuan Agricultural University.

### 4.2. Population Construction and Phenotypic Investigation

Two synthetic hexaploid wheat lines were sown in the greenhouse in July 2020, and Syn-SAU-117/Syn-SAU-119 F_1_ plants were subsequently generated. These F_1_ seeds were sown in a greenhouse in March 2021. Syn-SAU-117/Syn-SAU-119 F_2_ seeds were sown in the greenhouse in July 2021. Syn-SAU-117/Syn-SAU-119 F_2:3_ plants were sown in the field in November 2021. Each plant was 10 cm apart within rows, 30 cm apart between rows, and 1.5 m in length. The stems were sampled according to the method of Kong et al. [11]. More than ten stems from the main tiller were randomly selected after flowering and were cross-sectionally cut at the center of each internode. The level of stem solidity was rated as 1–5 (1 for hollow and 5 for solid) following Pauw et al. [34].

### 4.3. Observation of the Anatomical Structures of Stems

The internodes were numbered consecutively from the base to the top of the stem. At the jointing stage, the main tiller was selected. The center of the second internode of the wheat stem base was cut into 1 cm pieces and then soaked in FAA fixative for more than 24 h [43]. The samples were sent to Wuhan CVI Biotechnology Co., Ltd. (Wuhan, China) (https://www.servicebio.cn/ accessed on 10 July 2022) for the preparation of paraffin sections. CaseViewer 2.3 (https://www.3dhistech.com/solutions/caseviewer/ accessed on 20 July 2022, Budapest, Hungary) was used to view the results of the paraffin section analysis.

### 4.4. Solid Stem Gene Expression Analysis

A quantitative reverse transcription polymerase chain reaction (qRT-PCR) system (Bio-Rad, Shanghai, China) was used to analyze the gene expression of *TdDof* [13]. The D1 probe primers (D1_F: GTTCCTGCACGCCATGGAC; D1_R: TCCCCCATCGTCGCCATTA) were specifically designed to distinguish differences in expression levels between different plants, and the housekeeping gene GAPDH was used as a reference for gene expression analysis. The main stems of three plants were sampled at Zadoks Stage 32 and Zadoks Stage 34 when the first two and four nodes were present on the stem. Approximately 0.5 cm of the stem was sampled, measuring from the bottom of the lowermost node toward the uppermost node. Total RNA extraction was performed by using the Tiangen DP441 RNA prep Pure RNA Extraction Kit according to the manufacturer’s protocol. The quality of RNA was assessed by using polyacrylamide gel electrophoresis, and RNA reverse transcription was performed by using the Fermentas K1622 RT Reverse Transcription Kit (Thermo Scientific, Waltham, MA, USA).

### 4.5. Bulked Segregant RNA-Seq (BSR-Seq)

Solid and semisolid RNA pools for RNA-Seq were constructed by using the F_2_ generations with different stem solidity in the greenhouse. Equal amounts of RNA from 20 homozygous solid-stemmed and 20 semisolid-stemmed generations were pooled to conduct bulked segregant analysis [71]. The RNA samples were sequenced on the platform of Chengdu Tiancheng Future Technology Co., Ltd. (https://www.tcuni.com/, accessed on 27 December 2021). Sequence quality control was performed by using the fastp software v0.19.5 [72]. RNA reads of the solid stem and semisolid stem bulks were aligned to the reference genome sequence of Chinese Spring v1.1 [13] and *Ae. tauschii* AL8/78 v4.0 by using the STARv2.5.1b software [73]. The unique and confident alignments were applied to call SNP variants by using the GATK v3.6 software [74]. The SNP variants with *p-values* from Fisher’s exact test (FET) of <1 × 10^−8^ and an allele frequency difference (AFD) of >0.6 were considered to be associated with solid stem suppression and further used as templates for developing SNP markers [71].

### 4.6. Kompetitive Allele-Specific PCR (KASP) Assays

The solidity-related SNPs and the 500 bp flanking sequences were used to design the KASP primers and test polymorphisms on the parental lines and the solid and semisolid stem DNA bulks. Polymorphic markers that could be reliably scored were genotyped on the F_2_ population of Syn-SAU-117 × Syn-SAU-119. For each KASP assay, a 10 µL reaction volume containing 5 µL of 2 KASP master mix (Biosearch Technologies, Shanghai, China), 1.4 µL of primer mix (mixture of 0.168 µM each forward A1 and A2 primers and 0.42 µM of reverse primer), 100 ng of genomic DNA, and 2.6 µL of ddH_2_O was prepared. The CFX96Touch™ real-time PCR detection system (Bio-Rad, USA) was used for amplification under the following conditions: 15 min at 94 °C, 10 touchdown cycles of 20 s at 94 °C, 60 s at 65–57 °C (decreasing by 0.8 °C per cycle), and 32 cycles of 20 s at 94 °C, followed by 60 s at 57 °C.

### 4.7. Data Analysis

Chi-square *(χ*^2^) tests were used to determine the goodness of fit for the observed segregation and expected ratios of the F_2_ and F_2:3_ populations. Linkage analysis was performed by using MAPMAKER/EXP v3.0b [75]. The Kosambi function was used to convert recombination values into genetic distances [76]. A logarithmic odds (LOD) ratio of 3.0 and a maximum distance of 50.0 cM were set as thresholds for the declaration of linkage. The genetic linkage map was drawn by using the Mapdraw v2.1 software [77].

### 4.8. Candidate Gene Analysis

The sequences of the *KASP-669* and *KASP-1055* markers linked to *Su-TdDof* were used for BLAST against the genomes of Chinese Spring v1.1 [13] and *Ae. tauschii* AL8/78 v4.0 [78]. Gene annotations between the flanking markers of the two genomes were retrieved from the Ensembl Plants (http://plants.ensembl.org/index.html, accessed on 12 November 2022) and Swiss-Prot (http://www.gpm-aw.com/html/swi-ss-prot.html, accessed on 12 November 2022) databases. Furthermore, the differentially expressed genes within the interval were screened and analyzed based on the results of RNA-seq with the screening criteria of FDR < 0.05 and |LogFC| > 1. Collinearity analysis was performed on the differentially expressed genes related to the function of solid stems among parents and mixed pools.

## Figures and Tables

**Table 1:** Genetic analysis of solid stem suppressor genes in the F_1_, F_2_, and F_2:3_ families of Syn-SAU-117 × Syn-SAU-119.

Parents and Cross	Generation ^a^	No. of Plants/Families	Observed Ratio ^b^			Actual Ratio	Expected Ratio	*χ* ^2^	*p-Value*
			S	Seg	Ss				
Syn-SAU-117	P_Ss_	20			20				
Syn-SAU-119	P_S_	20	20						
P_Ss_ × P_S_	F_1_	20			20				
	F_2_	156	36		120	0.9:3	1:3	0.308	0.579
	F_2:3_	134	35	67	32	1.04:2:0.96	1:2:1	0.134	0.935

^a^ P_Ss_: semisolid parent Syn-SAU-117; P_S_: solid parent Syn-SAU-119. ^b^ S: homozygous solid; Seg: segregating within F_2:3_ families; Ss: homozygous semisolid.

**Table 2:** Primer sequences of KASP markers used for the genetic mapping of *Su-TdDof*.

Marker	Physical Position (bp)	Allele 1 Primer ^a^	Allele 2 Primer ^b^	Common/Reverse Primer
*KASP-533*	5,336,907	TCAGCTTCAATTTCGGCAGC	TCAGCTTCAATTTCGGCAGT	AGAAGCTGAACGTGCGGAAG
*KASP-669*	6,695,986	GTCGGATTCGGTTACTTTGAC	GTCGGATTCGGTTACTTTGAT	AGAGGTGCATGGTGTCGT
*KASP-1055*	10,558,194	TCTTTCTCCTTCAGCCTCTTA	TCTTTCTCCTTCAGCCTCTTG	GCCTGATTGTAGTACATTATG
*KASP-1166*	11,664,145	AACGAGGTCCCGCGCTCCTCCC	AACGAGGTCCCGCGCTCCTCCG	GTGTGAAGAGCGCTTCTGC

^a^ A1 primer labeled with FAM: GAAGGTGACCAAGTTCATGCT; ^b^ A2 primer labeled with HEX: GAAGGTCGGAGTCAACGGATT.

**Table 3:** Genotyping of AS92, Syn-SAU-117, Syn-SAU-119, and AS96 by using KASP markers linked to *Su-TdDof*.

Parents	Marker Genotype ^a^			
	*KASP-533*	*KASP-669*	*KASP-1055*	*KASP-1166*
AS92	CC	CC	AA	CC
Syn-SAU-117	CC	CC	AA	CC
Syn-SAU-119	TT	TT	GG	GG
AS96	TT	TT	GG	GG

^a^ AA, CC, GG, and TT represent the haplotype results of SNP genotyping.

## Data Availability

The data presented in this study are available on request from the corresponding author.

## Supplementary Materials

The following supporting information can be downloaded at: https://www.mdpi.com/article/10.3390/ijms241612845/s1.
