# Supplementary figures and images for: Identification of the Solid Stem Suppressor Gene Su-TdDof in Synthetic Hexaploid Wheat Syn-SAU-117
Source: Int J Mol Sci. 2023 Aug 16;24(16):12845. doi: 10.3390/ijms241612845 (PMC10454891; doi:10.3390/ijms241612845)

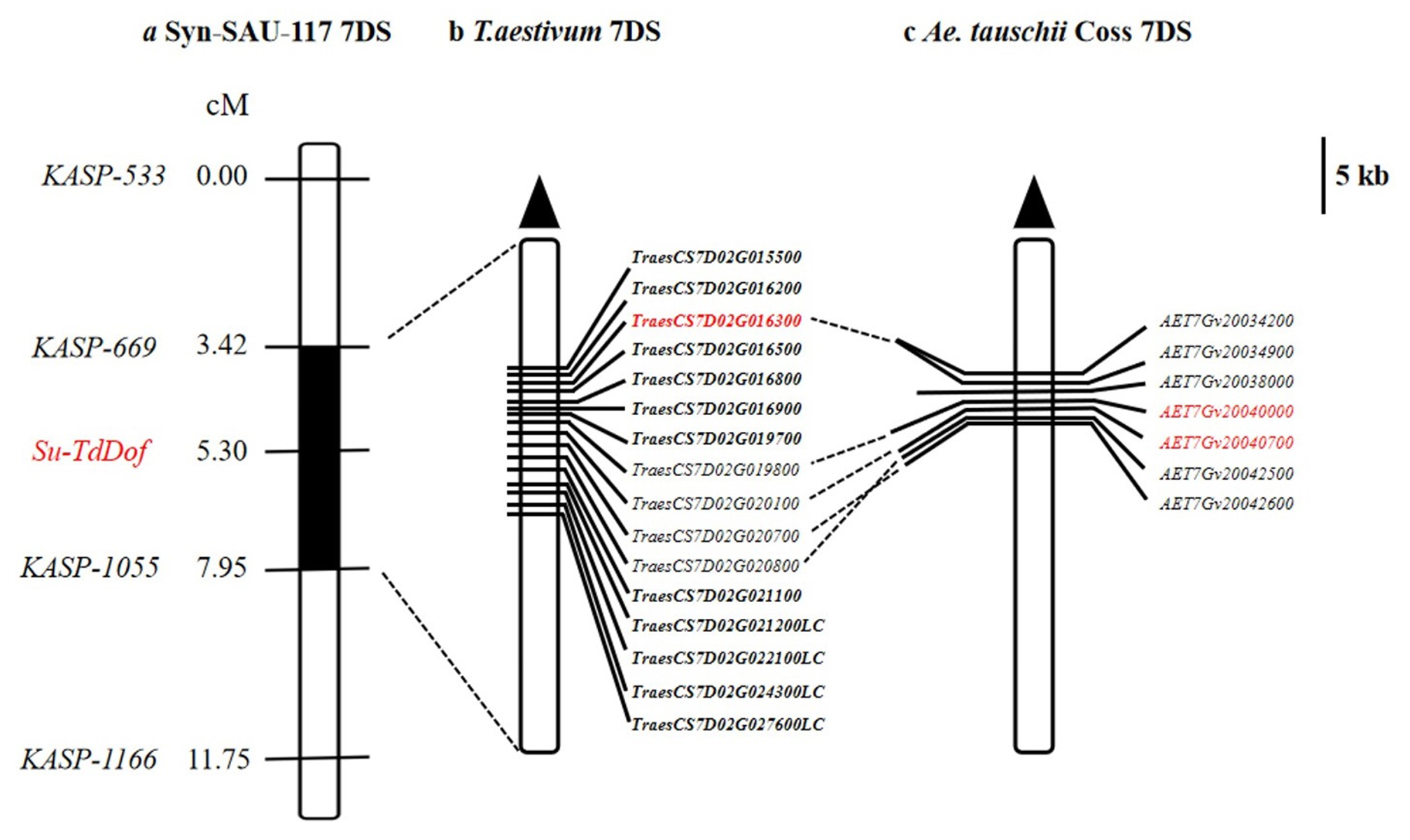

Supplement: Supplementary file 1 [file ijms-24-12845-s001.zip › Figure S1.jpg]
